# Influence of upper-body continuous, resistance or high-intensity interval training (CRIT) on postprandial responses in persons with spinal cord injury: study protocol for a randomised controlled trial

**DOI:** 10.1186/s13063-019-3583-1

**Published:** 2019-08-13

**Authors:** David W. McMillan, Jennifer L. Maher, Kevin A. Jacobs, Armando J. Mendez, Mark S. Nash, James L. J. Bilzon

**Affiliations:** 1 The Miami Project to Cure Paralysis, University of Miami Miller School of Medicine, Miami, FL USA; 2 Department of Kinesiology and Sport Sciences, University of Miami, Miami, FL USA; 3 Division of Endocrinology, Diabetes and Metabolism, University of Miami Miller School of Medicine, Miami, FL USA; 4 Department of Neurological Surgery, University of Miami Miller School of Medicine, Miami, FL USA; 5 Department of Physical Medicine and Rehabilitation, University of Miami Miller School of Medicine, Miami, FL USA; 6 Department for Health, University of Bath, Bath, Somerset UK

**Keywords:** Exercise, Spinal cord injury, Upper-body exercise, Continuous resistance training, High-intensity interval training, Postprandial, Metabolism

## Abstract

**Background:**

Chronic spinal cord injury (SCI) increases morbidity and mortality associated with cardiometabolic diseases, secondary to increases in central adiposity, hyperlipidaemia and impaired glucose tolerance. While upper-body Moderate Intensity Continuous Training (MICT) improves cardiorespiratory fitness, its effects on cardiometabolic component risks in adults with SCI appear relatively modest. The aim of this study is to assess the acute effects of Continuous Resistance Training (CRT), High Intensity Interval Training (HIIT), MICT and rest (CON) on fasting and postprandial systemic biomarkers and substrate utilisation.

**Methods:**

Eleven healthy, chronic SCI (> 1 year, ASIA A-C) men will be recruited. Following preliminary testing, each will complete four experimental conditions, where they will report to the laboratory following an ~ 10-h overnight fast. A venous blood sample will be drawn and expired gases collected to estimate resting metabolic rate (RMR). In order to ensure an isocaloric exercise challenge, each will complete CRT first, with the remaining three conditions presented in randomised order: (1) CRT, ~ 45 min of resistance manoeuvres (weight lifting) interspersed with low-resistance, high-speed arm-crank exercise; (2) CON, seated rest; (3) MICT, ~ 45 min constant arm-crank exercise at a resistance equivalent to 30–40% peak power output (PPO) and; (4) HIIT, ~ 35 min arm-crank exercise with the resistance alternating every 2 min between 10% PPO and 70% PPO. After each ~ 45-min condition, participants will ingest a 2510-kJ liquid test meal (35% fat, 50% carbohydrate, 15% protein). Venous blood and expired gas samples will be collected at the end of exercise and at regular intervals for 120 min post meal.

**Discussion:**

This study should establish the acute effects of different forms of exercise on fasting and postprandial responses in chronic SCI male patients. Measures of glucose clearance, insulin sensitivity, lipid and inflammatory biomarker concentrations will be assessed and changes in whole-body substrate oxidation estimated from expired gases.

**Trial registration:**

ClinicalTrials.gov, ID: NCT03545867. Retrospectively registered on 1 June 2018.

**Electronic supplementary material:**

The online version of this article (10.1186/s13063-019-3583-1) contains supplementary material, which is available to authorized users.

## Background

Chronic spinal cord injury (SCI) increases morbidity and mortality associated with cardiovascular [1] and metabolic diseases [2]. These clinical outcomes are preceded by a higher prevalence of known risk factors including central adiposity [3], hyperlipidaemia [4] and impaired glucose tolerance [5]. These elevated risks are not restricted to persons with SCI, but also affect the broader disabled population. Persons with a range of physical disabilities demonstrate a 1.2- to 3.9-fold higher prevalence of obesity than those without a physical disability [6]. It seems intuitive to suggest that this elevated prevalence of cardiometabolic component risks is, at least in part, associated with the Disability-Associated Low Energy Expenditure Deconditioning Syndrome (DALEEDS), first described by Rimmer et al. [7].

### Therapeutic benefits of exercise training

Traditional forms of Moderate Intensity Continuous Training (MICT) can provide some therapeutic benefits and reduce some components of cardiometabolic risk in persons with SCI [8]. Sixteen weeks of upper-body MICT, with minimal resistance training, reduces total body fat mass and visceral adipose tissue content in persons with SCI [9]. While just 6 weeks of MICT can also enhance fasting markers of hepatic insulin sensitivity in persons with paraplegia, the effects on postprandial markers of peripheral insulin sensitivity are negligible [10]. Indeed, neither of these training studies were able to demonstrate a significant benefit of MICT on cardiometabolic components related to postprandial insulin sensitivity, hyperlipidaemia or systemic inflammation. This has led to a call for high-quality randomised controlled trials to assess the efficacy of higher-intensity forms of exercise and assess the acute post-exerciseresponses in systemic biomarkers and energy homeostasis and to novel exercise challenges [11]. Currently, relatively little is known about the acute regulation of energy homeostasis at rest, during or post exercise in the SCI population.

### Acute metabolic responses to exercise in SCI

When compared to non-injured controls performing voluntary leg exercise, persons with SCI have markedly reduced mobilisation, delivery, and limb uptake of free fatty acids (FFA) during electrically stimulated leg exercise [12]. This is most likely the result of reduced sympathoadrenal β-adrenergic stimulation and/or limited neural activity in motor centers and afferent nerves from contracting skeletal muscles, depending on the level of injury [12]. The limited availability of FFA during exercise leads to a heavy reliance on carbohydrate (CHO), with a concomitant limited contribution from fat as a fuel source [13, 14]. These observations appear consistent across a variety of modes and intensities of exercise [15, 16]. Increased reliance on carbohydrate as a fuel source during exercise may affect the metabolic handling of systemic glucose and lipids, both during and post exercise.

### Energy expenditure in persons with SCI

In non-injured individuals, human energy expenditure (EE) is elevated during recovery from exercise [17]. These increases in EE occur in a dose-dependent manner, which is predominantly related to exercise intensity and modality, as opposed to exercise duration [17]. Only three studies have compared changes in EE following isocaloric arm and leg cycling in non-injured humans and have demonstrated only modest increases in post-exercise EE in response to arm cycling [18–20]. Arm cycling resulted in a lower cumulative post-exercise EE compared to isocaloric leg cycling [19], suggesting that upper-extremity exercise per se has a limited ability to elevate post-exercise EE. Only one study has examined these responses to arm cycling in persons with SCI [21], demonstrating only a modest increase in post-exercise EE, which was similar to that observed in non-injured humans. This may reflect a reduced ability of the upper-body skeletal muscles to tolerate sustained anaerobic metabolism and accumulate oxygen debt. However, it remains to be determined if exercise mode and/or intensity have the ability to modulate post-exercise EE in persons with SCI.

### Substrate metabolism in persons with SCI

Associated with the increases in EE at rest, both CHO [22] and fat oxidation rates [23–25] are significantly increased during recovery from exercise. This effect of exercise on substrate partitioning occurs in a manner dependent more on the total energy cost of exercise and less on exercise intensity or metabolic rate [23]. While the increase in post-exercise fat oxidation has been demonstrated in non-injured humans [20], to date, no studies have examined this response in persons with SCI. Our pilot data [26] demonstrate that fat oxidation is elevated for at least 120 min following ~ 50 min of circuit resistance training (Continuous Resistance Training; CRT) in persons with SCI.

### Postprandial responses in persons with SCI

Several studies in persons with SCI have reported exaggerated postprandial lipaemia (PPL) [27, 28] and glycaemia (PPG) [29], which is of some concern, as such chronically elevated responses are component risks for cardiometabolic disease [30]. In persons without SCI, a juxta-meal exercise bout has a dramatic effect on the metabolic handling and disposal of macronutrients from a meal. Specific to CHO and fat disposal, pre-meal exercise attenuates PPL [25] and PPG [22] even in persons with known disorders of energy homeostasis [22, 29]. Interestingly, both exercise modality [17, 24] and intensity [17, 31] have been shown to modify the effect of exercise on post-exercise energy homeostasis, independent of exercise-related EE. However, to our knowledge, no studies have examined the acute effects of different forms of exercise on postprandial systemic metabolite, hormone or biomarker concentrations or whole-body substrate oxidation rates in persons with SCI.

## Objectives

In summary, it is currently unknown whether, in persons with SCI, the mode or intensity of pre-meal exercise influences the metabolic handling and oxidation of macronutrients. The objectives of the proposed study are, therefore, to compare the effects of resting as control (CON), Moderate Intensity Continuous Training (MICT), High Intensity Interval Training (HIIT) and Continuous Resistance Training (CRT) on:

1. Fasting systemic concentrations of metabolites, hormones and relevant inflammatory biomarkers
2. Postprandial systemic concentrations of metabolites, hormones and relevant inflammatory biomarkers
3. Postprandial EE and whole-body substrate oxidation rates

The central hypotheses were that higher-intensity, intermittent upper-body exercise (i.e. HIIT and CRT) will enhance measures of fasting and postprandial insulin sensitivity and fasting triglyceride concentrations, compared to moderate-intensity exercise (MICT) or rest (CON).

## Methods/design

### Study design

**Fig. 1:**
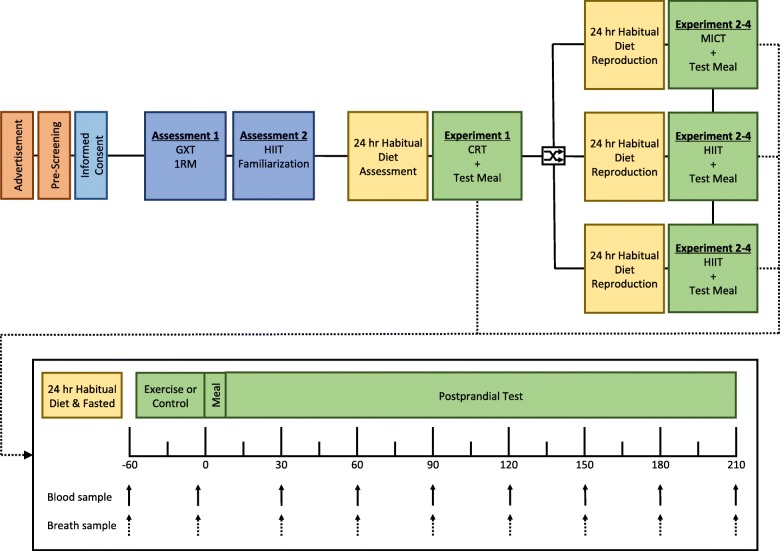
Study flow diagram demonstrating the order of recruitment, testing and main trial procedures

This study is a partially randomised, repeated-measures, counter-balanced, crossover design. Participants will attend two preliminary sessions including baseline assessments and a HIIT familiarisation session before completing the four experimental conditions. Each participant will complete the CRT condition first in order that the intensity and/or duration of the HIIT and MICT protocols can be adjusted to deliver an isocaloric exercise challenge. The CON, MICT and HIIT conditions will be completed in a randomised order, at least 3 days apart. The planned experimental design is summarised below (Fig. 1) and is consistent with current Standard Protocol Items: Recommendations for Interventional Trials (SPIRIT) guidelines [32, 33]. The study protocol has been approved by the Human Subjects Research Office, Miller School of Medicine, University of Miami (Institutional Review Board No. 20171114, Version 3, dated 5 February 2018) and the trial has been registered as a current controlled trial (ClinicalTrials.gov, ID: NCT03545867 on 1 June 2018).

### Study setting

All human testing will take place in the Lois Pope Life Center at the Miami Project to Cure Paralysis at the University of Miami’s Miller School of Medicine. All biochemical analyses will take place at the Diabetes Research Institute, Division of Endocrinology, Diabetes and Metabolism at the University of Miami Miller School of Medicine.

### Recruitment

It has been reported that participant recruitment to research studies, especially in the absence of direct access to a clinical population, requires considerably more resources and time than initially anticipated in order to achieve adequate enrolment [34]. Patient barriers are reported to include [35]: (1) additional demands of the trial increasing participant burden and (2) patient preferences for or against a particular treatment. Barriers to research participation are perhaps even more exaggerated for people with disabilities due to complex health problems [36], lack of transportation [37], cognitive impairments and financial stress [38].

In order to overcome some of these barriers and challenges, The Miami Project maintains and supports the running of a clinical exercise programme, where individuals with SCI who have volunteered to participate in a Miami Project study, can access a fully equipped gymnasium free-of-charge. Associated with this, a user database is maintained, containing basic descriptive information on persons with SCI who have volunteered to participate in previous studies and volunteered ongoing participation. Upon initial contact with individuals from this user community (i.e. email or telephone), the study will be described and those interested in enrolling will be asked to read a detailed participant information sheet and complete a health screening questionnaire. Interested individuals will be screened via a telephone conversation 48 h after their initial contact to ascertain eligibility. A focus will be placed on establishing a good rapport at this initial contact and also as participants progress through the enrolment process (Fig. 1). Written consent will be obtained during the initial laboratory visit and participants will be informed that they can withdraw from the study at any time without consequence. Participants will be paid for their participation in this study, following completion of all assessments. This is primarily designed to reimburse travel expenses and thank them for any inconvenience caused.

### Randomisation

After completion of baseline laboratory testing, the HIIT familiarisation and the CRT condition, participants will complete the remaining three experimental conditions in random order. Randomisation will be performed by an independent senior researcher (MN) using a list generated with a web-based platform (http://www.randomization.com), concealed from those involved in participant management (DM, JM and JB) to prevent biased allocation [39, 40].

### Participants

To address our hypothesis, a cohort of 10 healthy men with chronic SCI, who satisfy the inclusion and exclusion criteria outlined below, will be recruited via advertisement and direct contact with the local SCI community.

Inclusion criteria: (1) Male; (2) Aged ≥ 18 years old; (3) Neurologically stable SCI (ASIA Impairment Scale A-C) at T6 and lower spinal levels for > 1 year; (4) Able and willing to comply with study procedures; (5) Have the ability to understand written and spoken English; and (6) Have the capacity to provide informed consent.

Exclusion criteria: (1) Female; (2) Aged < 18 years; (3) Contraindication to exercise (ACSM Guideline, 10th edition); (4) Lower-extremity fracture or dislocation within 6 months of participation; (5) History of head injury or seizures; (6) Inability to provide informed consent; (7) Restrictions in upper-extremity range of motion that would prevent an individual from achieving an unhindered arm cycling motion or moving throughout a range needed to perform resistance manoeuvres; (8) Had a pressure ulcer at the ischial/gluteus, trochanteric, sacral or heel sites within the last 3 months; (9) Imprisonment in state or federal jail or prison; (10) Taking any medication that might interfere with the study outcomes (this will be reviewed by the research team on a case-by-case basis should a potential participant be on regular medication. The *British National Formulary* will be checked for potential effects that might introduce bias in the study); and (11) Illness/condition that might interact with study measures (e.g. diabetes, heart disease) or pose undue personal risk.

### Preliminary assessments

#### General

Participants’ cardiorespiratory fitness and muscular strength will be assessed using the procedures outlined below. Their descriptive characteristics (i.e. age, body mass) and basic injury characteristics will be recorded via self-report questionnaire. Following these assessments, the participants will schedule their remaining laboratory test days (outlined below). They will be instructed on how to record their normal daily diet on a ‘food log’ that will be completed the day prior to the first main trial (i.e. CRT). Before each of the following three trial days, participants will be provided with (1) a copy of their original food log and (2) a blank food log, and will be asked to reproduce this dietary intake to the best of their abilities, but to record on the new food log their actual dietary intake.

#### Arm-crank ergometry (ACE) cardiorespiratory fitness testing

Cardiorespiratory fitness (V̇O_2_ peak) will be assessed during a graded exercise test (GXT). Participants will refrain from exercise/alcohol/caffeine for 24 h prior to testing and perform a continuous GXT on an electrically braked arm-crank ergometer (Lode Angio, Groningen, Netherlands) at a constant cadence of 60 ± 5 rpm. A digital display will provide real-time feedback on cadence. Every 3 min, resistance will increase by 20 W (W). Participants will continue until volitional exhaustion manifesting as either a non-verbal communication of the desire to stop or an inability to maintain cadence above 55 rpm. Upon cessation, participants will rest quietly for 10 min. Prior to each test, a staff member will interview the participant to determine the individualised wattage starting work rate and increments to target a V̇O_2_ peak (i.e. volitional exhaustion) within 9–15 min. Starting loads will range from 0–50 W, with smaller loads for sedentary individuals. This approach is consistent with the American College of Sports Medicine’s Guidelines for Exercise Testing and Prescription (10th edition) recommendation that starting loads and increments be individualised to the participant’s perceived functional capacity [41]. Expired gases will be continuously collected in a Hans-Rudolph Softmask worn by the participant and analysed by a portable open-circuit indirect calorimetry system (Oxycon Mobile, Viasys, Inc., Conshohocken, PA, USA).

#### One-repetition maximum (1-RM) muscular strength testing

To determine the resistance levels assigned for the CRT trial, upper-extremity dynamic strength testing will be performed on a Helms equaliser 1000 multi-station exerciser (Helm Distributing, Polson, MT, USA) using the following manoeuvres: overhead press, horizontal row, vertical butterfly, biceps curl, latissimus pull down (either to the chest or neck) and dips. Participants will be instructed to perform eight repetitions of each manoeuvre, with each repetition lasting 6 s (3 s concentric, 3 s eccentric). If eight repetitions are completed in controlled fashion, the weight will be increased and the exercise repeated. Incremental increases in weight will be provided until eight controlled repetitions cannot be completed. The 1-repetition maximum (1-RM) will be calculated using previously published equations [42]:

1 $$ 1\hbox{-} RM= WT/\left( 0.533+ 0.419E\hbox{-} 0.055\ast REPS\right) $$

where ‘1-*RM*’ is the calculated 1-repetition maximum strength, ‘*WT*’ is the resistance used in the last set where more than three, but fewer than eight repetitions are completed, and ‘REPS’ equals the number of repetitions completed in the last set of testing.

### HIIT familiarisation

During their second preliminary visit to the laboratory, participants will be fitted with a Hans-Rudolph Softmask and expired gases will be collected and analysed throughout exercise (as described above). Participants will conduct ~ 50 min of arm-crank ergometry (ACE) on the same device/position as described above during the GXT. The cycle ergometer will be programmed to vary power output so that a warm-up and cool-down (2 min) and the active recovery intervals will be completed at 10% peak power output (PO_peak_), and the working intervals completed at 70% PO_peak_. The ratio of work:recovery intervals will be 1:1. The EE data will later be used to calculate the duration of HIIT required to elicit an isocaloric challenge to CRT.

### Main trials

**Fig. 2:**
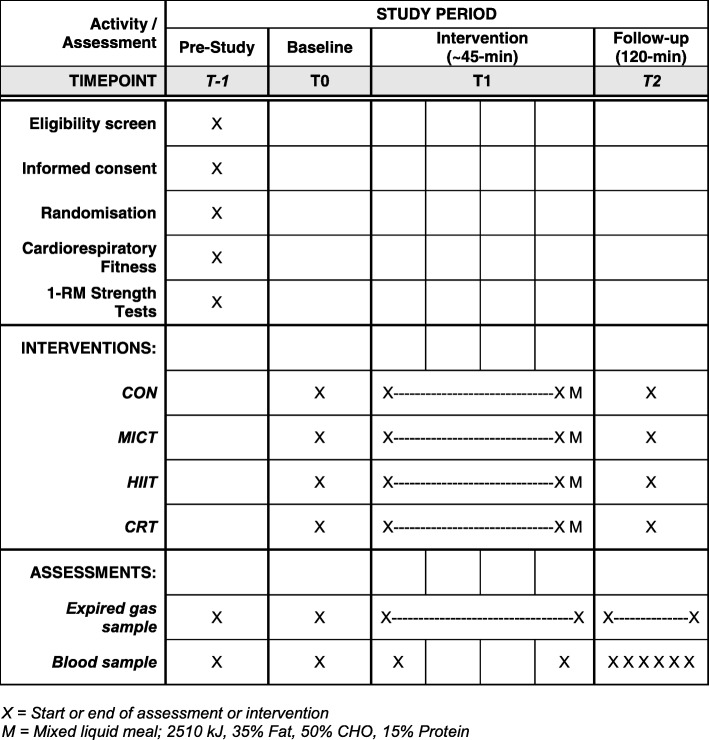
Standardised Protocol Items: Recommendations for Interventional Trials (SPIRIT) Figure

The same experimental procedures will be completed on all four main trial days (Fig. 1). These procedures are also reflected in the Standardised Protocol Items: Recommendations for Interventional Trials (SPIRIT) Figure (Fig. 2). The associated SPIRIT Checklist is available online (Additional file 1). Twenty-four hours prior to each main laboratory trial, participants will abstain from caffeine (tea/coffee) and alcohol. On the morning of the main trials, participants will be instructed to consume ~ 10 ml.kg^− 1^ of water on waking and report to the laboratory at 0800 hours ± 0.5 h following an overnight fast (≥ 10 h). Following entry to the laboratory participants will be fitted with the mask for indirect calorimetry (as described above) and will remain seated prone in their wheelchair for ~ 10 min to assess resting EE (REE). Immediately after this, an initial 10-ml venous blood sample will be drawn. For the next ~ 50 min, expired gases and heart rate values will continue to be collected while the participants rest (CON) or exercise (MICT, HIIT or CRT). Immediately after this period, an indwelling cannula will be inserted in to an antecubital vein and kept patent with sterile saline. An initial sample will be drawn before participants consume a 600-kcal liquid meal test meal (35% fat, 50% CHO, 15% protein). Further 10-ml venous blood samples will be drawn at 0, 15, 30, 60, 90 and 120 min post meal. Expired gases will continue to be collected throughout the postprandial period.

#### Resting control (CON)

During the resting control (CON) condition, participants will remain seated in a prone position for 50 min. If they require the bathroom during this period, they will be pushed to and from the room and the time recorded.

#### Moderate Intensity Continuous Training (MICT)

**Fig. 3:**
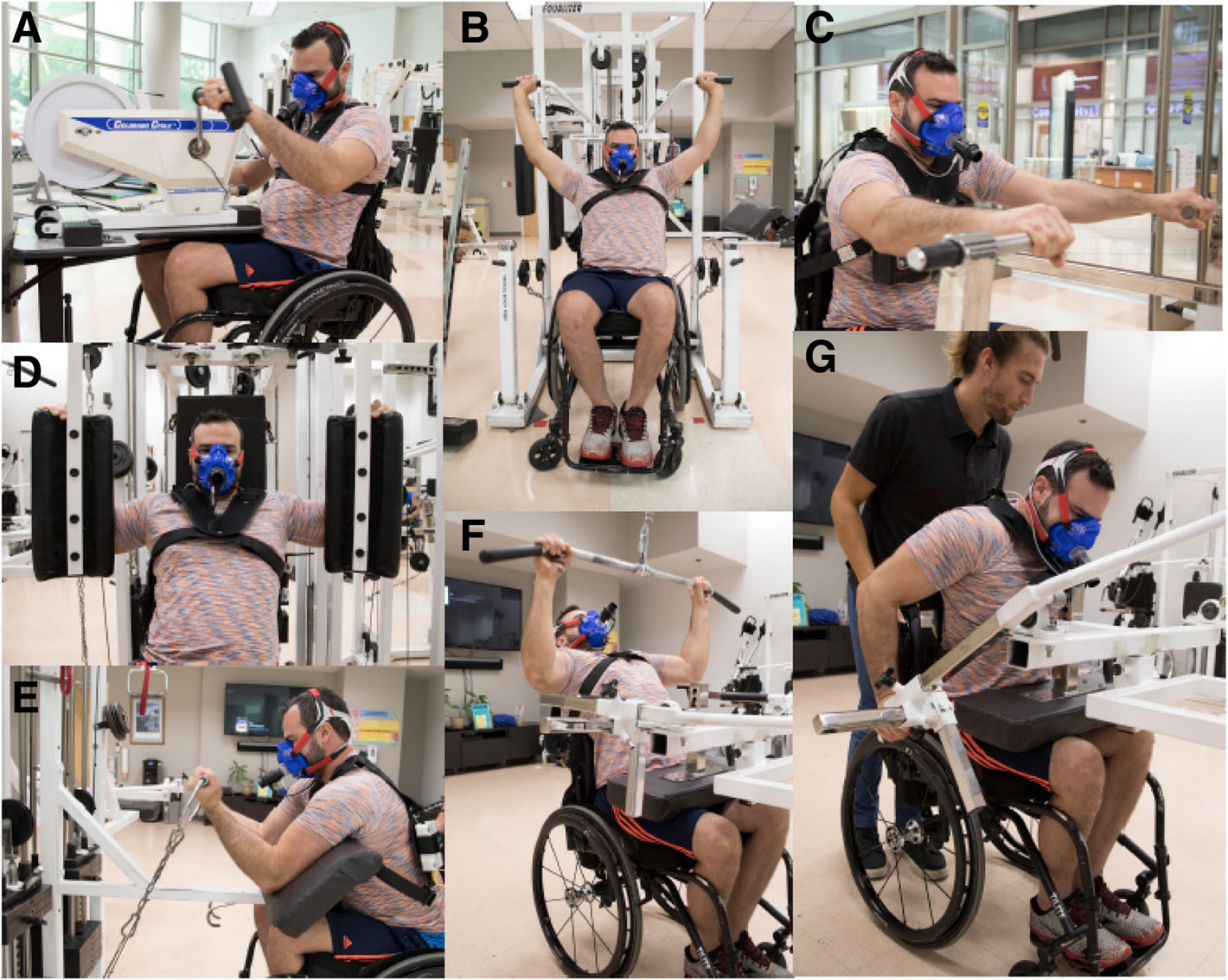
Images of a single participant completing the various elements of the exercise testing regimen: **a** arm-crank ergometry. **b** military press. **c** horizontal row. **d** pectoralis (‘pec’) deck. **e** preacher curl. **f** wide-grip latissimus pull-down. **g** seated dip

Following baseline measurements, participants will conduct ~ 50 min of ACE on the same device/position as described above during the same electronically braked ergometer (Fig. 3a). The cycle ergometer will be programmed to provide a constant predetermined resistance equivalent to ~ 60% of the PO_peak_ achieved during the GXT. The exact duration of the MICT trial will be calculated to deliver an isocaloric challenge that is equivalent to the CRT trial.

#### High Intensity Interval Training (HIIT)

Following baseline measurements, participants will conduct ~ 50 min of ACE on the same electronically braked ergometer (Fig. 3a). The cycle ergometer will be programmed to vary the resistance to produce a power output for the warm-up, cool-down (2.5 min) and active recovery intervals equivalent to 10% PO_peak_, and the working intervals completed at 70% PO_peak_. The ratio of work:recovery intervals will be 1:1 and HIIT will continue until an estimated (from the HIIT familiarisation trial) total EE equivalent to the CRT condition is achieved.

#### Continuous Resistance Training (CRT)

Following baseline measurements, participants will conduct ~ 50 min of CRT consisting of resistance manoeuvres (weight lifting) and low-resistance, high-speed endurance activities (arm-cranking). The efficacy of the CRT for delivering fitness and health-related benefits for persons with paraplegia have been described previously [43–45] and recently adopted by the Paralyzed Veterans of America and endorsed by the member organisations of the American Spinal Injuries Association [46]. Participants will perform 10 repetitions of lifting on each of the following exercise stations: (1) military press (Fig. 3b), (2) horizontal rows (Fig. 3c), (3) pectoralis (‘pec’) deck (Fig. 3d), (4) preacher curls (elbow flexion) (Fig. 3e), (5) wide-grip latissimus pull-down (Fig. 3f) and (6) seated dips (Fig. 3g). All manoeuvres will be conducted at 60% 1-RM as determined during strength testing. Every time participants complete two resistance exercises they will perform low-resistance, high-speed arm exercise for 2 min on a stationary cycle. They will rest for 10 s between each set of repetitions, and will complete three cycles of the exercises. Our previous data indicate that CRT will elicit a mean exercise EE of ~ 170 kcal in persons with paraplegia [26]. However, the individual variability is large (SD = 55 kcal), primarily dependent on the resistance set for each activity, hence the need to conduct CRT before the other exercise interventions.

### Emergencies and adverse events

Participants will be monitored for the following both during and after treatments: headaches, pain, lightheadedness, dizziness, altered vision, respiratory distress, cyanosis, spasms, or change in function. We will also monitor cardiovascular responses to treatment to ensure that participants do not exceed lower (< 40 bpm) and upper (> 180 bpm) limits for heart rate or systolic blood pressure (i.e. < 85 mmHg and > 200 mmHg).

The skin located near pressure points will be inspected after every exercise session. Exercise trials will be stopped if the participants experience any of the following: chest pain, dyspnoea, diaphoresis, or a pale or ashen appearance. All testing is being performed at a medical centre and is, therefore, located near an emergency room. Security personnel at the Miami Project are all trained ‘first responders’. The laboratory has approved policies and procedures for emergencies and all staff have current certification for cardio-pulmonary resuscitation.

All adverse events will be reported to the Institutional Review Board (IRB) within the mandated time period. For any adverse event the principal investigator will immediately notify and consult with the study physician, who will come to an opinion as to whether the event(s) is/are related to the study procedures described in the protocol. Adverse events will be evaluated by the study physician using the following criteria:

i.) Grade 1 (mild): awareness of symptoms, but easily tolerated; usually transient requiring no special treatment; does not interfere with usual or normal daily activities
ii.) Grade 2 (moderate): may be ameliorated by simple therapeutic measures; may interfere with, but not keep the participant from participating in normal daily activities
iii.) Grade 3: incapacitating event, inability to perform usual activities
iv.) Grade 4 (life-threatening/disabling): patient is at risk of death, or worsening disability or impairment as existed at the time of the event

For the first two grades the investigators will observe the participant and as necessary institute standard medical or therapeutic care. Repeated occurrence of grade 1 and 2 events may be cause for notification of the IRB by the investigators that stoppage is appropriate. The investigators may take this action and the IRB will be so notified. Grade 3 and 4 events will be individually evaluated. Any occurrence of grade 3 and 4 events may be cause for notification of the IRB by the investigators that stoppage is appropriate. The investigators may take this action and the IRB will be so notified. Otherwise, upon on an IRB determination that the serious adverse event was related to the protocol, the study will stop and undergo evaluation for continuation.

### Experimental measures

#### Energy expenditure

Energy expenditure and substrate oxidation rates will be determined from expired gas analysis, at rest and during exercise, using indirect calorimetric methods. The following equations will be used [47]:

2 $$ Energy\ expenditure\ \left( kcal/\mathit{\min}\right)\kern0.5em =\kern0.5em 3.941\cdot \overset{.}{V}{O}_2+1.106\cdot \overset{.}{V}{CO}_2 $$

3 $$ CHO- ox\ \left(g/\mathit{\min}\right)\kern0.5em =\kern0.5em 4.344\cdot \overset{.}{V}{CO}_2-3.061\cdot \overset{.}{V}{O}_2 $$

4 $$ Fat- ox\ \left(g/\mathit{\min}\right)\kern0.5em =\kern0.5em 1.695\cdot \overset{.}{V}{O}_2-1.701\cdot \overset{.}{V}{CO}_2 $$

Pre-exercise REE will be accepted as stable when repeated measurements are within 100 kcal/^.^day [48, 49] and the lowest of these measures will be accepted as RMR [50].

#### Blood sampling

Following the ~ 50 min rest or exercise sessions, a cannula (BD, Venflon™ Pro, Becton Dickenson & Co., Stockholm, Sweden) will be inserted into an antecubital vein from which repeated 10-ml blood samples will be drawn, as previously described. Plasma samples will be centrifuged immediately at 3466 g at 4 °C for 10 min (Heraeus Biofuge Primo R, Kendro Laboratory Products Plc., Tyne and Wear, UK). Serum samples will be left to clot for 60 min at room temperature before centrifugation. All samples will be dispensed into 1.0-ml aliquots and stored at − 80 °C.

### Outcome measures

In order to simplify data analysis and facilitate the interpretation of a complex dataset [51, 52], serial measurements of glucose and insulin responses at baseline, post exercise and in response to the rest/exercise challenge will be converted into simple summary statistics [53], such as incremental area under the curve (iAUC) [54] and insulin sensitivity index (ISIMatsuda) [55]. The Homeostasis Model Assessment (HOMA) calculator, incorporating the updated HOMA-2 model [56], will be used to derive fasting estimates of pancreatic β-cell function, insulin resistance and sensitivity, both at rest and post exercise. A full lipid profile (i.e. triglycerides, total cholesterol, non-esterified fatty acids (NEFA), high-density lipoprotein cholesterol (HDL-C), low-density lipoprotein cholesterol (LDL-C)) will also be assessed to establish the influence of exercise protocol on post-exercise and postprandial responses. From indirect calorimetric measurements, EE, fat and carbohydrate oxidation rates will be determined and compared, primarily during the postprandial phase. The primary and secondary outcome variables are presented in Table 1.

**Table 1:** Primary and secondary outcome variables

Outcome measure	Analytical method
Metabolites	
Glucose	Plasma glucose concentration will be assessed using an automated analyser (Randox RX Daytona, Co. Antrim, UK).
Lipids	Serum markers of total triglycerides, total cholesterol, NEFA, HDL-C and LDL-C will also be assessed using an automated analyser and commercially available immunoassays (Randox Laboratories, Co. Antrim, UK).
Hormones	
Insulin	Determined using ELISA (Mercodia AB, Uppsala, Sweden). Variables: (i) incremental area under the curve (iAUC) [54]; (ii) insulin sensitivity index (ISIMatsuda) [55]; (iii) Homeostasis Model Assessment (HOMA-2) model [56]
Inflammatory markers	
Il-6	Baseline and post-exercise measures of IL-6 will be assessed using ELISA (Quantikine HS, R&D Systems Inc., Abingdon, UK)
Energy expenditure	
Total energy expenditure (kcal/min)	Total energy expenditure and substrate oxidation rates will be determined from expired gas analysis, at rest and during exercise, using indirect calorimetric methods, as previously described [47]
CHO-ox (g/min)	
Fat-ox (g/min)	

Abbreviations: *CHO* carbohydrate, *ELISA* enzyme-linked immunosorbent assay, *HDL-C* high-density lipoprotein cholesterol, *IL* interleukin, *LDL-C* low-density lipoprotein cholesterol, *NEFA* non-esterified fatty acids

### Data storage and availability

#### Electronic data records

All electronic files are stored on password-protected computers in rooms 1–48 and 1–50 of the Lois Pope Life Center. Computer security is provided by data encryption, firewall protection, and data backup on the Miami Project server. Source data obtained from metabolic analysis will be entered into a de-identified data bank and stored securely on the network.

#### Physical data records

Data will be stored in a locked room (LPLC 1–50), in a locked file cabinet, which is only accessible to study personnel in the Lois Pope Life Center, 1095 NW 14th Terrace, Miami, FL 33136, USA. The metabolic analyser used to obtain data is located locked in a room (1–49) accessible by university ID badge after passing a security desk and a hallway accessed by proximity card entry. A security badge is needed to open the office door where the data are stored. A security guard is located in the front lobby, and the hallway to the storage site is secured by proximity card entry.

#### Procedures to release data

To maximise access to the final dataset, after publication of the analyses described within this protocol, we will deposit the final dataset in a to-be-determined data repository. Prior to deposit in the repository, the final dataset will be anonymised. Access to data and procedures to release the data will be defined by the policies of the repository in which the data are placed.

### Power calculation

We plan to recruit 11 male participants with chronic (> 1 year) SCI, by engaging users from the Miami Project to Cure Paralysis community. The primary outcome variables are related to fasting and postprandial glycaemic control, but specifically postprandial insulin area under curve (iAUC). While there are only two previous studies that have compared the effects of MICT and HIIT on biomarkers of metabolic regulation in non-disabled humans [57, 58], both suggest no effect of MICT (vs. CON) and an enhanced effect of HIIT. We therefore hypothesised that there will be no differences in postprandial glycaemic control between CON and MICT and a similarly enhanced effect of HIIT and CRT. Using data from these previous studies, the mean predicted effect size for postprandial iAUC is ~ 1 [57] and for postprandial triglyceride concentrations is 0.97 [59]. We therefore estimated that a final sample size of 11 will be required for this repeated measures study, to provide approximately 90% power to detect a significant difference in ∆ insulin iAUC at an alpha level of 0.05. Recruitment will proceed on a rolling basis until the adequate sample size is reached; emphasis will be placed on considering the demands of the study before enrolling to reduce drop-out during the study.

### Statistical analysis

All data from participants who successfully complete the study will be included in the analysis. The differences in key outcome variables between experimental conditions (CON, MICT, CRT and HIIT) and time (dependent on variable) will be analysed using a two-way (condition × time) fully repeated measures analysis of variance (ANOVA). Where significant interactions are observed, multiple *t* tests will determine the location of variance. Significant post-hoc effects will be subjected to a Holm-Bonferroni stepwise adjustment. Standardised effect sizes (Cohen’s *d*) will also be calculated. This will provide a practical interpretation of the size of the effects of each experimental condition relative to CON. For all the above statistical approaches, statistical significance will be set at an alpha level of *p* ≤ 0.05. We will explore the use of confidence intervals and magnitude-based inferences to assess the clinical significance of the effect.

## Discussion

Previous research suggests that higher-intensity exercise, resulting in greater skeletal muscle glycogen depletion within an individual session, is likely necessary to enhance biomarkers of cardiometabolic component risk, particularly peripheral insulin sensitivity [43, 57, 58] and postprandial triglyceride concentrations. This may be particularly true in humans that have lost the ability to innervate a large proportion of their skeletal muscle mass, such as persons with SCI. Such conditions create numerous barriers to physical activity engagement and the need to maximise the potential metabolic benefits of individual bouts of physical activity becomes paramount. This has led to a recent call for high-quality randomised controlled trials to assess the efficacy of higher-intensity exercise protocols on cardiometabolic component risks in persons with SCI [11]. However, before engaging in such complex intervention studies, it is important to understand the acute physiological and metabolic responses to a single bout of different forms of upper-body exercise in this population.

To our knowledge, this study will be the first to compare the acute metabolic responses of persons with SCI to two novel, higher-intensity modes of upper-body exercise (CRT and HIIT) with a more traditional form of exercise (MICT) and rest (CON). While the two forms of higher-intensity exercise are quite different in nature, they both include intermittent exposures to higher-intensity metabolic activity, which is likely to lead to a relatively greater level of muscle glycogen depletion than MICT [60]. As muscle glycogen concentration is an important driver of acute changes in peripheral insulin sensitivity following exercise, this presents a very plausible mechanism for at least an acute up-regulation of glycaemic regulation [61–63]. This approach will help elucidate the exercise modalities and associated mechanisms, which will likely provide the greatest therapeutic stimulus for enhanced physiological function and metabolic regulation in a population with a high prevalence of cardiometabolic disease.

## Trial status

Institutional Review Board Protocol No. 20171114, Version 3, dated 5 February 2018. Recruitment start date: 1 March 2018. Recruitment end date: 4 July 2018.

## Additional file

Additional file 1: Standard Protocol Items: Recommendations for Interventional Trials (SPIRIT) 2013 Checklist: recommended items to address in a clinical trial protocol and related documents. (DOC 121 kb)

## Data Availability

To maximise access to the final data, at the point of publication, we will deposit the final dataset in a public data repository. Prior to deposit in the repository, the final dataset will be anonymised. Access to data and procedures to release the data will be defined by the policies of the repository in which the data are placed.

## Abbreviations

ACE: Arm-crank ergometry
ASIA: American Spinal Injuries Association
CHO: Carbohydrate
CON: Resting control condition
CRIT: Continuous, Resistance or High Intensity Interval Training study
CRT: Continuous Resistance Training
DALEEDS: Disability-Associated Low Energy Expenditure Deconditioning Syndrome
FFA: Free fatty acids
GXT: Graded exercise test
HIIT: High Intensity Interval Training
iAUC: Incremental area under the curve
MICT: Moderate Intensity Continuous Training
PPG: Postprandial glycaemia
PPL: Postprandial lipaemia
PPO: Peak power output
REE: Resting energy expenditure
RMR: Resting metabolic rate
SCI: Spinal cord injury
V̇O_2_ peak: Peak oxygen uptake
